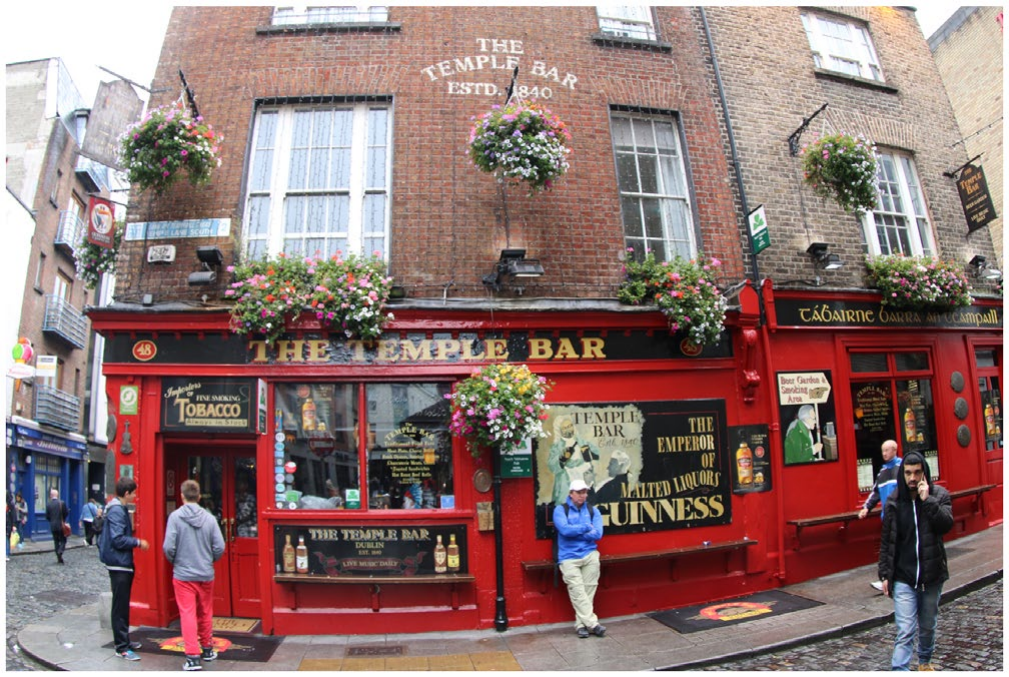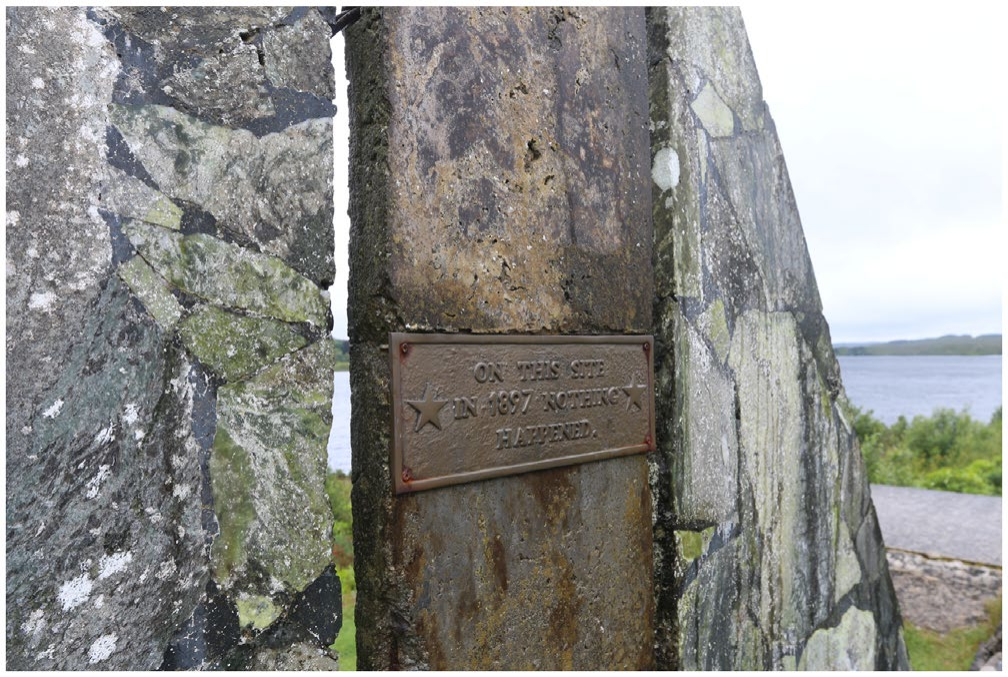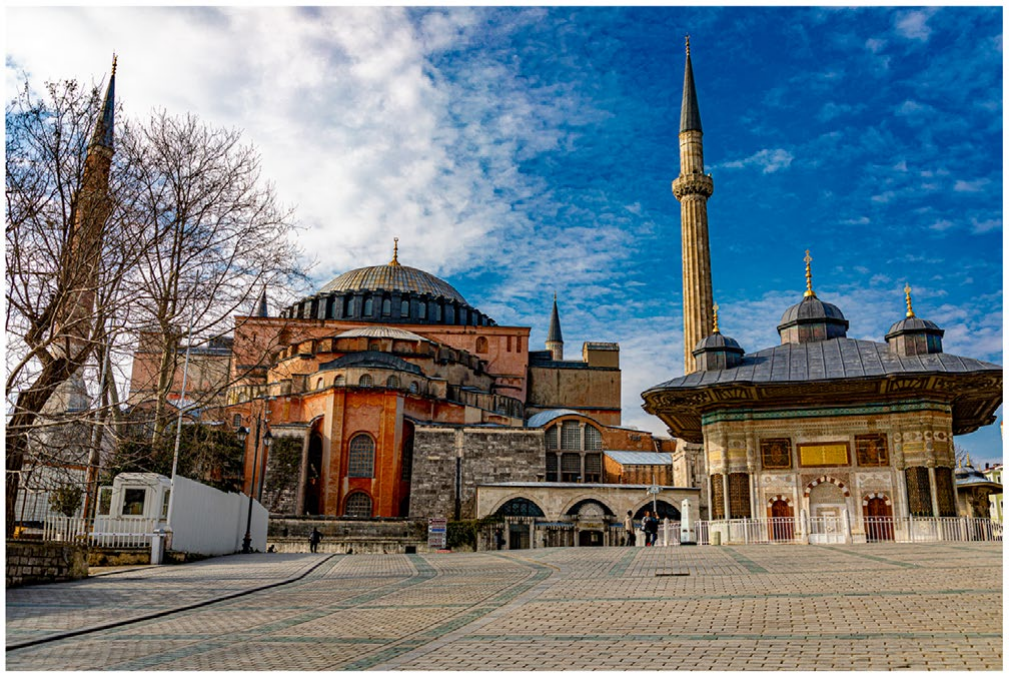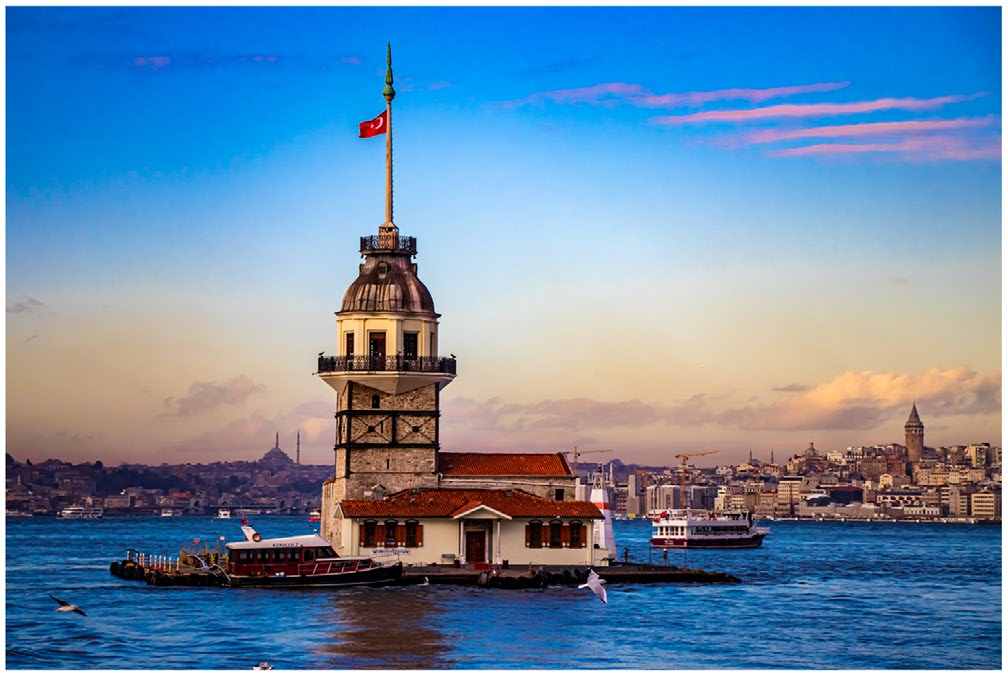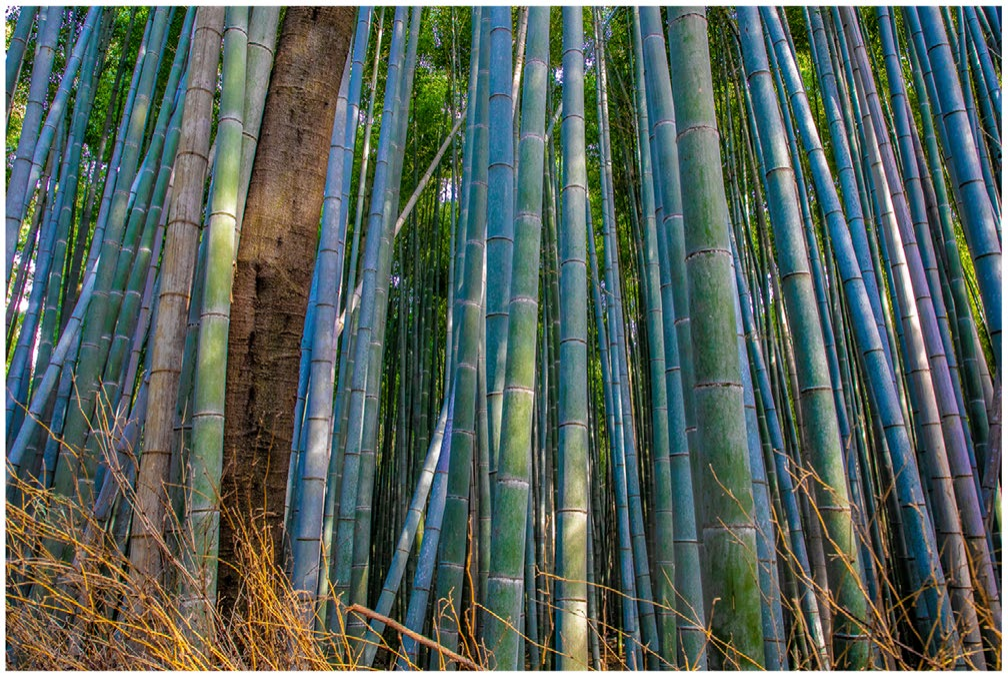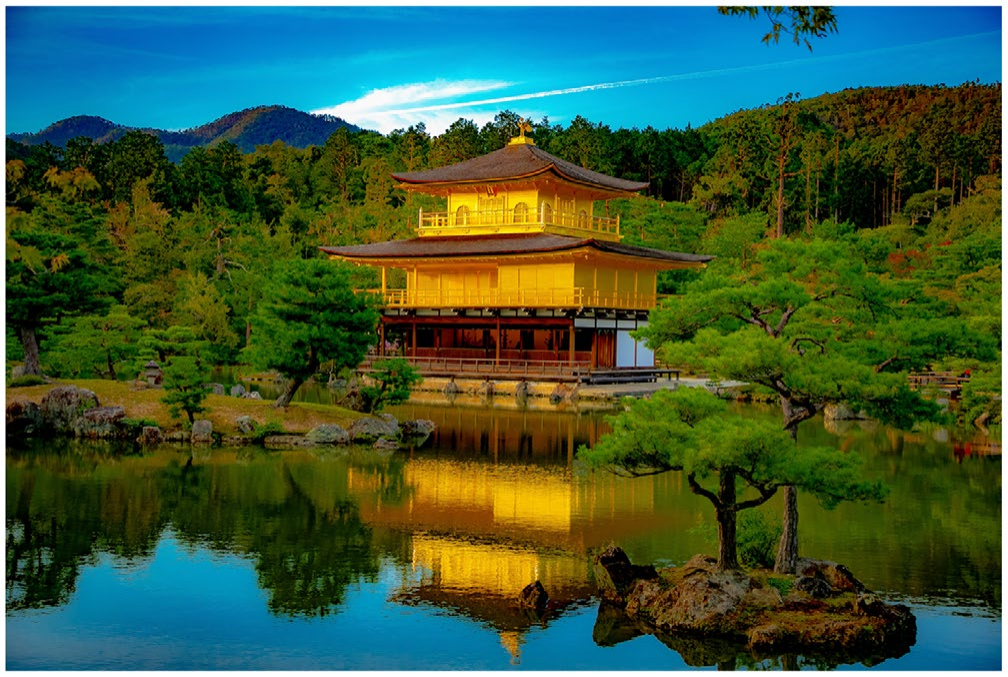# Four directions and four continents

**DOI:** 10.1002/bco2.30

**Published:** 2020-07-13

**Authors:** John W. Davis

**Affiliations:** ^1^ BJUI Compass

For our third issue of *BJUI Compass*, I am pleased to see a recent increase in paper submissions—ideas discussed months ago that are now finalized and into peer review, transfers from the *BJUI*, and direct submissions. For the remainder of 2020 and into 2021, we will keep to our four‐direction theme, with at least four articles, and publication every other month. I want to thank our editorial board for contributing as authors, reviewers, social media outreach, and idea generation.

Of course, one topic among many on everyone’s mind is the evolution of the Covid‐19 pandemic and how we will ever get out of this. At the time of this writing, I am in my home state of Texas, which is one of several populated states to experience an upsurge in Covid‐19 cases since “opening for business” a few weeks ago after many weeks of stay‐at‐home orders. The next few weeks will be critical to determine whether or not we can function with open economies yet contain the disease through social distancing (shouldn’t the term be physical distancing?), hand washing, and case containment. Many are on edge for another surge and strain on hospital resources.

As a result, online learning continues while in‐person meetings continue to be canceled or converted to virtual formats. Just this weekend I participated in two virtual activities. The *Indian Journal of Urology* and Urological Society of India put on a weekend webinar on presentation and publication skills—a very concise 2‐h program with three lectures, faculty discussion, and several hundred participants joining through YouTube and Zoom interfaces. Another virtual meeting—and a first‐time experience for many of us—was the recent graduation ceremony for the University of Texas at Houston Urology Residency. In lieu of a nice banquet at a local restaurant, we had to go with WebEx. Yet most of the key elements of the event could be preserved: junior residents roasting the graduating chiefs, videos of thanks and congratulations from faculty and staff, a program director’s words, and speeches from the graduates. So as this is our July issue when many training programs are turning over, let me pass on my congratulations to all for the many milestones achieved in our field—joining us as trainees for the first time, moving up to the next level, or graduating to join our work force.

Moving to the July 2020 *BJUI Compass* issue, we have four more interesting articles for our readers.

- *To the Journals…* The paper by Brooks et al continues our interest in Covid‐19 related articles. As you may recall, the May issue featured a nice review by Badar Mian on the initial response to Covid‐19 with Twitter related discussions on how to respond, followed by what has come out in the literature, and international perspectives. The Brooks paper takes the discussion into the academic realm of what we should be doing about the challenge. They center upon the fascinating theme of whether or not a common urologic drug like BCG can be re‐purposed for Covid‐19 prevention. The basic observation that crude case fatality rates appear lower in countries with BCG vaccination has led to a global effort to study this question in clinical trials—many active and enrolling already. The authors give a nice global perspective on the mechanism and uses of BCG. I have heard of similar hypotheses for the protective effects of androgen deprivation therapy. Hopefully these efforts along with vaccine research will help us make progress with this deadly disease.
- *To the Clinic…* Lima et al from the University of Miami give a very practical report on their series of microsurgical varicocelectomy and ask the question whether or not the hormonal levels are improved to correlated with improvements in semen parameters. The results, at the hormone sub‐type level, were negative as outlined, and what ensues is a discussion on how/why semen parameters change and what mechanisms are causal.
- *To the Drawing Board…* Academic innovation often requires authors to dive into datasets and to look for novel predictors for key outcomes. In the context of surgical therapy for prostate cancer, the paper by Momota et al from Japan focused on the outcome of pain experiences after robotic prostatectomy. The key finding was that frailty was significantly associated with moderate‐to‐severe pain after surgery. Using the frailty scales the authors suggest higher risk patients for alternate pain management pathways. In my personal practice, anesthesia delivers a post‐induction TAP block (transversus Abdominis Plane) with bupivacaine to smoothen out post‐op pain issues and try to achieve same day discharge for single port cases, and early next AM discharge for multi‐port cases. You can also refer back to our March 2020 issue paper by Janet Kukreja for a more comprehensive review of ERAS protocols (enhanced recovery after surgery).
- *To the Future…* On any short list of “future” concepts in urologic surgery would be imaging guided surgery. Canda et al from Istanbul, Turkey present a short series of how this process might work—pretreatment three‐dimensional MRI and PSMA‐PET scan, intraoperative imaging with augmented reality images for real tie tumor navigation, and follow‐up histopathological mapping. Many of these individual tools are utilized and have their own body of literature, and these authors put the ingredients together nicely and show a pathway toward improving the accuracy of tumor eradication and avoiding positive surgical margins.

Figure 1: The BJUI Compass is an international journal (the I in BJUI) and welcomes all countries to contribute. In the July 2020 issue, we have papers with authorships from the United States, Ireland, Turkey, and Japan—some of my favorite places to visit.

1a: The iconic Temple Bar in Dublin with your editor holding the wall up.

1b: On the road from Galway to Roundstone, one of the more memorable plaques I’ve seen. Do read the details. Is this Irish humor?

1c: The famous Ayasofya museum in Istanbul’s Old Town.

1d: Maiden’s Tower, Istanbul—built on a small islet at the Southern entrance of the Bosphorous strait.

1e: The Arashiyama Bamboo Grove, Kyoto, Japan—so many shades of green with sunbeams trying to break in.

1f: A must see tourist destination—Kinkakuji, Golden Pavilion, Kyoto—a Zen temple covered in gold.